# Changes in the gut microbiome due to diarrhea in neonatal Korean indigenous calves

**DOI:** 10.3389/fmicb.2025.1511430

**Published:** 2025-03-05

**Authors:** Ji-Yeong Ku, Mi-Jin Lee, Youngwoo Jung, Hak-Jong Choi, Jinho Park

**Affiliations:** ^1^Department of Veterinary Internal Medicine, College of Veterinary Medicine, Jeonbuk University, Iksan, Republic of Korea; ^2^Department of Veterinary Nursing, College of Health Science, Wonkwang University, Iksan, Republic of Korea; ^3^Technology Innovation Research Division, World Institute of Kimchi, Gwangju, Republic of Korea

**Keywords:** neonatal Korean indigenous calves, gut microbiome, diarrhea, diversity, taxonomic composition, linear discriminant analysis effect size

## Abstract

Studies on gut microbiome changes in neonatal Korean indigenous calves with diarrhea are rare. In this study, 14 normal calves and 11 calves with diarrhea were selected from Korean indigenous calves up to 30 days of age and classified into three groups at 10-day intervals (1–10, 11–20, and 21–30 days). Feces from 25 calves were collected, and the diversity, similarity, structure, and correlation of the gut microbiome were analyzed. *Firmicutes*, *Bacteroidetes*, and *Proteobacteria* were predominant in the taxonomic composition of the gut microbiome of the calves regardless of the presence of diarrhea. However, *Proteobacteria* increased and *Bacteroidetes* and *Actinobacteria* decreased in calves with diarrhea. In addition, calves with diarrhea showed a significant decrease in the diversity of the gut microbiome, especially for anaerobic microorganisms *Faecalibacterium prausnitzii*, *Gemmiger formicilis*, and *Collinsella aerofaciens*. The microbial communities in calves with diarrhea and normal calves were distinct. By analyzing the microorganisms that showed correlation with diarrhea and age using linear discriminant analysis effect size, at the genus level, *Prevotella* and *Lachnospiraceae_uc* were significantly related in the normal (11–20 days) group whereas *Enterobacterales*, *Gammaproteobacteria*, *Enterobacteriaceae*, *Escherichia*, and *Proteobacteria* were significantly associated with diarrhea in the 11–20 days group. Futhermore, the normal (21–30 days) group showed significant correlation with *Blautia*, *Provotellaceae*, Muribaculaceae, Christensenellaceae, and Catenella, whereas the diarrhea (21–30 days) group showed significant correlation with *Dorea*. The microorganisms associated with diarrhea in calves were mainly known as harmful microorganisms, we confirmed that there is a relationship between the increase in harmful bacteria and diarrhea. These results show that diarrhea significantly affects the gut microbiome of Korean indigenous calves. The changes in the gut microbiome of Korean indigenous calves observed in this study could be helpful in predicting and managing diarrhea calves, and furthermore, in establishing preventive measures for calf diarrhea through management of gut microbiome.

## 1 Introduction

The gut microbiome plays an important role in maintaining host health. Cattle have a large microbial community from birth; however, the gut microbiome of neonatal calves, which has not yet been established, constantly changes due to various causes. The presence or absence of colostrum intake immediately after birth in calves affects the ratio of harmful bacteria in the gut microbiome, and the change in feed from milk to solid feed as the calves grow brings about a clear change in the gut microbiome (Hartinger et al., 2022; Song et al., 2019). In addition, the fecal microbiota of calves is affected by the type of feed and sex, which creates differences in the ratio of Firmicutes/Bacteroidetes related to feed efficiency (Sim et al., 2022). And the breeding environment can also affect the gut microbiome, such as grazing on pastures increases the diversity of the gut microbiome (Jung et al., 2022).

In calves, dysbiosis of the gut microbiome due to diarrhea or antibiotics affects host immunity, metabolism, and protection against pathogens (Khalil et al., 2022; Amin and Seifert, 2021). Additionally, a decrease in the abundance of microbial genes related to amino acid metabolism has been observed in calves with diarrhea (Gomez et al., 2017). The microbiota of newborn calves can have long-term effects on the health, of not only the calves but also the cattle (Kerr et al., 2015); previous study reported that differences in the gut microbiome existed between calves infected with bovine coronavirus and normal calves, even after recovery from diarrhea (Kwon et al., 2021). In addition, improving the microbiota through the intake of beneficial microorganisms and fecal microbiota transplantation is helpful for the growth and health of calves (Du et al., 2023; Kim et al., 2021). An increase in *Porphyromonadaceae* in the gut microbiome due to microbial transplantation improves diarrhea in Korean indigenous calves, and has a potential role in growth performance (Kim et al., 2021). Additionally, an increase in *Faecalibacterium* spp. in the gut microbiome of calves increases their body weight and reduces the incidence of diarrhea, whereas an increase in *Escherichia coli* is highly correlated with the occurrence of gastrointestinal diseases (Slanzon et al., 2022; Oikonomou et al., 2013).

Diarrhea in newborn calves is a major cause of mortality, and when it occurs, it causes many problems such as growth retardation, environmental contamination due to antibiotic use, and economic loss to livestock farms (Hur et al., 2013; Kim et al., 2015; Sischo et al., 1990; Urie et al., 2018; Wittum et al., 1993). Diarrhea is caused by a combination of infectious and non-infectious factors (Bendali et al., 1999; Frank and Kaneene, 1993; Lorenz et al., 2021; Windeyer et al., 2014). Newborn calves are at high risk of colonization by intestinal pathogens because of their immature gut microbiome, and diarrhea is more common (Caballero-Flores et al., 2023; Kim et al., 2017; Li et al., 2019; Pickard et al., 2017). Many studies have reported that diarrhea causes an imbalance in the gut microbiome, and that there are significant differences in the gut microbiome between healthy calves and calves with diarrhea (Oikonomou et al., 2013; Shi et al., 2023; Zeineldin et al., 2018; Schwaiger et al., 2022). In newborn calves, the diversity of the gut microbiome was significantly reduced after rotavirus infection, which causes diarrhea, and significant differences in the composition of the gut microbiome were confirmed compared to healthy calves (Kim et al., 2023; Cui et al., 2023; Jang et al., 2019). Previous study reported a significant decrease in *Lactobacilli* up to 24 h before the onset of diarrhea in newborn calves (Schwaiger et al., 2022). Despite significant changes in the gut microbiome of calves due to diarrhea, most studies on Korean indigenous calves are still focused on detecting the causative agents of diarrhea in calves, and studies related to changes in the intestinal environment of neonatal Korean indigenous calves due to diarrhea are still lacking (Kim et al., 2021; Chae et al., 2021; Kim et al., 2016; Ryu et al., 2020).

Domesticated Korean indigenous cattle, called Hanwoo, produce about 1 million calves annually. Korean indigenous cattle are mainly managed systematically in indoor farms, but digestive diseases in calves still occur frequently (Kim et al., 2015). The intestinal microbiota plays an important role in the health of calves, and understanding these microorganisms will aid in the management of the health of calves. However, since there are differences in the gut microbiome depending on the calf species, there is a lack of data on the gut microbiome of Korean indigenous calves. Furthermore, an appropriate method for managing the intestinal microbiota is needed. Therefore, this study investigated the effects of diarrhea on the gut microbiome of Korean indigenous calves of different ages. To this end, the diversity and structure of the fecal microbiota of Korean indigenous calves with and without diarrhea were analyzed to identify differences in the gut microbiome. The aim was to identify key microorganisms associated with diarrhea in calves that could be used as biomarkers for with efficient growth and management of the health of calves through early diagnosis and prevention of diarrhea.

## 2 Materials and methods

### 2.1 Ethics statement

This study was approved by the Institutional Animal Care and Use Committee of the National Institute of Animal Science, Republic of Korea (JBNU IACUC no. NON2023-123).

### 2.2 Animals

To determine the differences in the gut microbiome according to diarrhea, feces were collected from 14 normal calves and 11 calves with diarrhea (Jeonbuk, South Korea) and under 30 days of age. Based on the judgment of an experienced veterinarian, calves that had no clinical problems after birth and a fecal score of 0 (normal) were selected as normal calves, and calves with a fecal score of 2 (runny) or 3 (watery) were selected as calves with diarrhea (Larson et al., 1977). All calves with normal or diarrhea were born at similar times on the same farm, received colostrum immediately after birth, and raised under the same management practices. The calves were grouped according to age into as 1–10 days old (normal, *n* = 5; diarrhea, *n* = 3), 11–20 days old (normal, *n* = 4; diarrhea, *n* = 5), and 21–30 days old (normal, *n* = 5; diarrhea, *n* = 3) groups (Table 1).

**Table 1:** Information of 80 Korean indigenous calves involved in this study.

Fecal consistency	Age (day)	Diarrhea	Group	*n*	Total
Solid/Semi-solid	1 ~ 10	Normal	Normal (1 ~ 10)	5	14
	11 ~ 20		Normal (11 ~ 20)	4	
	21 ~ 30		Normal (21 ~ 30)	5	
Loose/Watery	1 ~ 10	Diarrhea	Diarrhea (1 ~ 10)	3	11
	11 ~ 20		Diarrhea (11 ~ 20)	5	
	21 ~ 30		Diarrhea (21 ~ 30)	3	

All feces were collected directly from the anus by wearing rectal examination gloves and massaging the rectal wall with fingers to relax it and encourage defecation. The collected feces were placed in sterile 50 mL tubes (Conical Tube, SPL Life Sciences, Pocheon, Korea) and transported to the laboratory under refrigeration. After dispensing 1 g of the fecal samples into 1.5 mL tubes (Axygen Scientific, Union City, CA, United States), they were stored at −20°C until analysis.

### 2.3 DNA extraction and polymerase chain reaction

The total genomic DNA of microorganisms present in the 25 fecal samples was extracted using a DNA extraction kit (FastDNA SPIN Kit for Soil, MP BIO) (Seethalakshmi et al., 2024).

The amplification and sequencing of the 16S rRNA gene of microorganisms extracted from each fecal sample was used as a template, and the V3/V4 region of the 16S rRNA gene was amplified using primers 341F and 805R and polymerase chain reaction (PCR) equipment (PTC-200 Peltier thermal cycler, MJ Research, Waltham, MA). The PCR conditions were pre-denaturation at 94°C for 3 min, followed by 28 cycles of denaturation at 94°C for 30 s, annealing at 53°C for 40 s, elongation at 72°C for 1 min, and then final extension at 72°C for 5 min. Secondary amplification for the attachment of the Illumina NexTera barcode was performed using the i5 forward and i7 reverse primers. The PCR conditions were pre-denaturation at 94°C for 3 min, followed by 8 cycles of denaturation at 94°C for 30 s, annealing at 53°C for 40 s, elongation at 72°C for 1 min, and then final extension at 72°C for 5 min. The primers and sequences used are listed in Table 2.

**Table 2:** Primers and gene sequences of polymerase chain reaction (PCR).

Primer	Sequence (5′ → 3′)
341F	TCGTCGGCAGCGTCAGATGTGTATAAGAGACAGCCTACGGGNGGCWGCAG
805R	GTCTCGTGGGCTCGGAGATGTGTATAAGAGACAGGACTACHVGGGTATCTAATCC
Illumina index i5 S502	ATGATACGGCGACCACCGAGATCTACACCTCTCTATTCGTCGGCAGCGTC
i7index i7 N701	CAAGCAGAAGACGGCATACGAGATTCGCCTTGTCTCGTGGGCTCGG

The amplified PCR products were purified using a QIAquick PCR Purification Kit (Qiagen, Valencia, CA, United States) and subjected to electrophoresis to select DNA with a sequence length of 300 bp or longer. DNA fragment lengths were determined using an Agilent 2,100 Bioanalyzer (Agilent Technologies, Santa Clara, CA, USA). A library was constructed using the amplified products, and sequencing was performed using MiSeq (Illumina).

### 2.4 Gut microbiome analysis

The base sequence data obtained from the MiSeq results were classified by sample using the Mothur software.^1^ The paired-end reads for each sample were then made into a single contig, and sequence filtering was performed to meet the criteria for quality control (Schloss et al., 2009). The filtered reads were analyzed for alpha diversity (operational taxonomic unit (OTU), rarefaction curve, Shannon-Wiener, Chao1, etc.), and microbial community structure and relationships at the phylum, class, order, family, genus, and species levels were identified using the EzBioCloud server^2^ and CL community (ChunLab Inc., Seoul, Republic of Korea). In addition, clustering was confirmed using the unweighted pair group method with arithmetic average (UPGMA), and beta diversity was measured by unweighted unique fraction metric (UNIFRAC) analysis (Lozupone et al., 2011; Clarke, 1993; Gower, 1971). Microbial changes were analyzed using principal coordinate analysis (PCoA) plots (Gower, 1966). The linear discriminant analysis effect size (LEfSe) method was used to identify bacterial taxa at *p* < 0.05, with and a linear discriminant analysis (LDA) score > 2.0, using the Galaxy workflow framework.^3^

### 2.5 Statistical analysis

The relative abundances of major phyla, classes, orders, families (median relative abundance >0.1%), and genera (median relative abundance >0.01%) were calculated, and comparisons between the groups were performed using GraphPad Prism 6 (GraphPad Software, Inc., La Jolla, CA, United States). Normality was analyzed using the Shapiro–Wilk test, and comparisons between two groups were made using the Mann–Whitney U test, and for three or more groups, the Kruskal-Wallis test. In all statistical analyses, the significance level was set at *p* < 0.05.

## 3 Results

### 3.1 Sequence reads

To identify differences in the intestinal microbiota according to age and the presence of diarrhea, 695,375 sequence reads were obtained from 25 fecal samples collected from Korean indigenous calves (average of 30,234 reads per calf) (Supplementary Figure 1).

### 3.2 Alpha diversity

**Figure 1:**
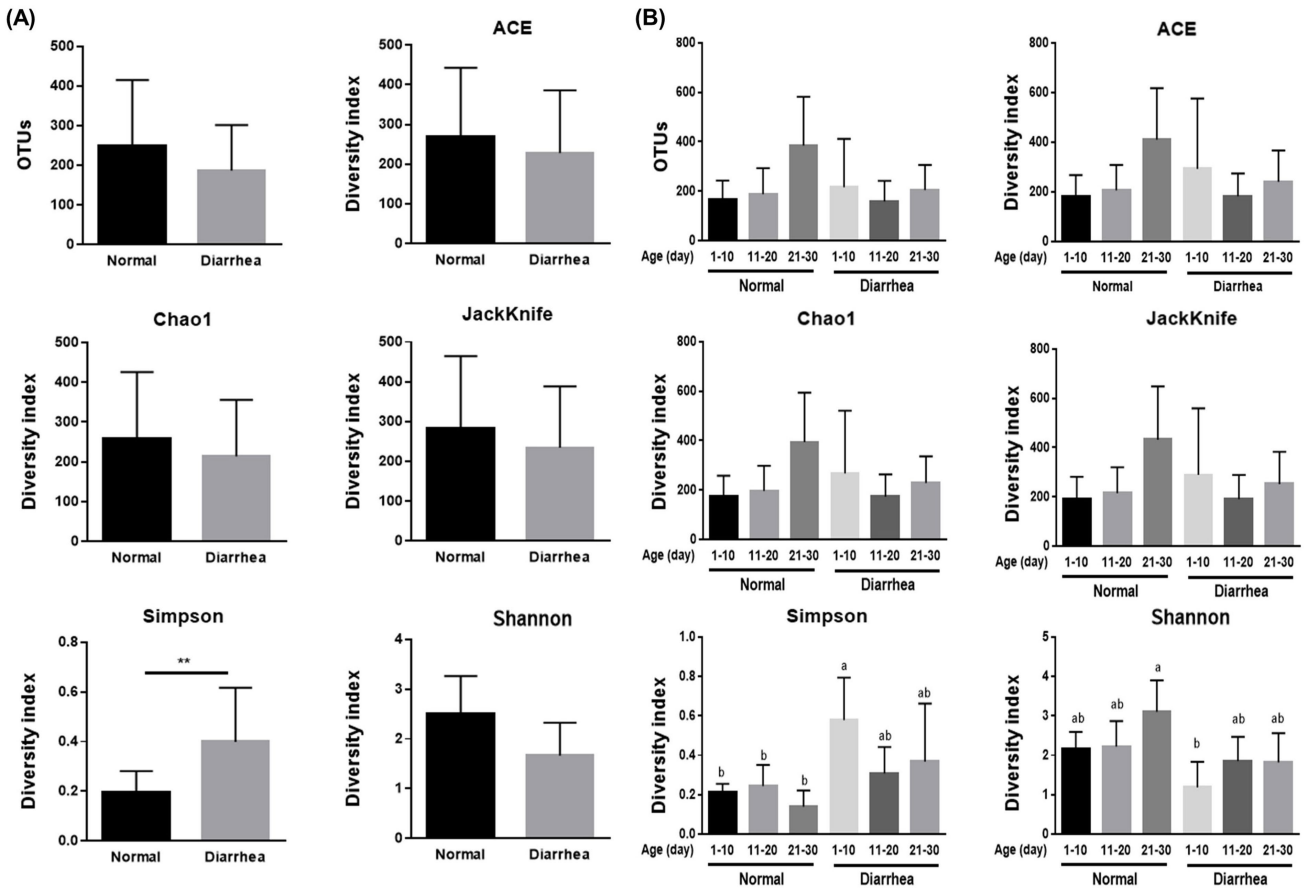
Results of alpha diversity analysis of the average gut microbiome **(A)** and gut microbiome according to age (1–10, 11–20, 21–30 days) **(B)** in normal calves (Normal) and calves with diarrhea (Diarrhea) within 30 days of age. ^**^p < 0.01, a,b; Same letters indicate no significant difference.

Depending on the presence or absence of diarrhea in the calves, the richness indices ACE, Chao1, and Jackknife decreased, but there was no significant difference. However, for the evenness indices, there was a significant increase in the Simpson index and a decrease in the Shannon index depending on the diarrhea (Figure 1). This suggests that diarrhea in calves causes a decrease in fecal microbiome diversity.

There was no significant difference in the richness indices of Ace, Chao-1, and Jackknife according to the age of the calves and the presence or absence of diarrhea. However, there was a significant difference in the Simpson evenness index between the normal (1–10 days) and diarrhea (1–10 days) groups and in the Shannon index between the normal (21–30 days) and diarrhea (1–10 days) groups (Figure 1).

### 3.3 Community membership and structure

**Figure 2:**
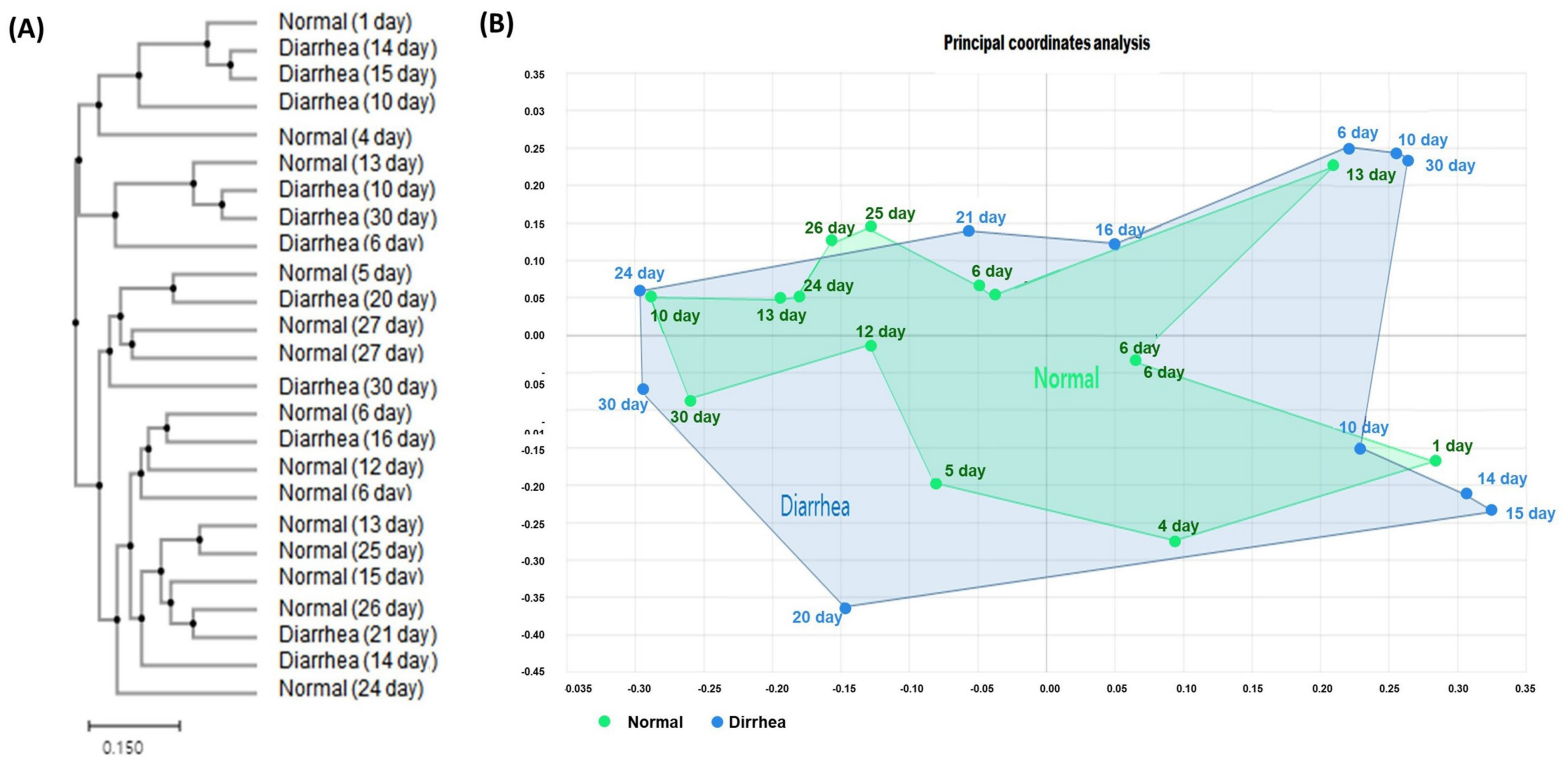
Results of clustering **(A)** and beta diversity **(B)** analysis of the gut microbiome of normal calves and calves with diarrhea within 30 days of age.

Clustering analysis and principal coordinate analysis (PCoA) were performed to determine the similarity relationship between the gut microbiota of calves. When categorized by the presence or absence of diarrhea in calves by age, calves with diarrhea showed distance differences in the gut microbiome compared with normal calves. However, the difference in distance between the normal (1–10 days) and diarrhea (1–10 days) groups was similar (Figure 2).

### 3.4 Taxonomic composition analysis

**Figure 3:**
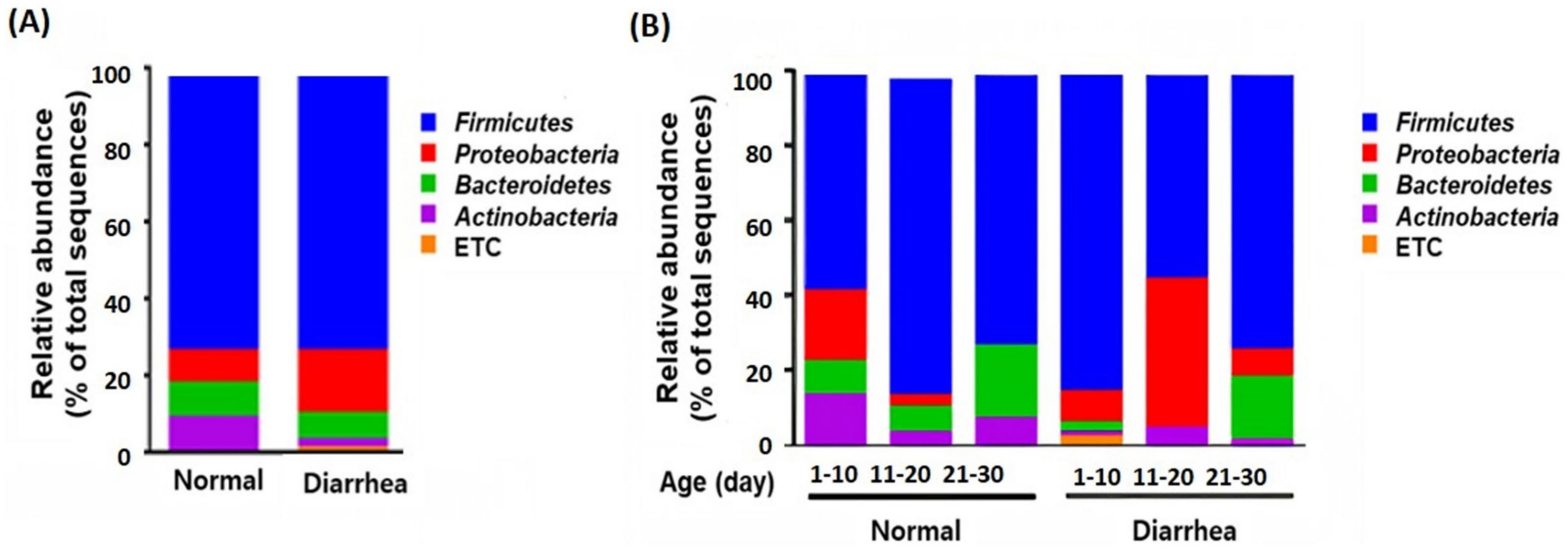
Taxonomic composition of the gut microbiota according to the average **(A)** and age group **(B)** of normal calves (Normal) and calves with diarrhea (Diarrhea) at the phylum level.

Analysis of the relative abundance at the phylum level between the groups according to the presence or absence of diarrhea showed that *Proteobacteria* increased and *Bacteroidetes* and *Actinobacteria* decreased in calves with diarrhea (Figure 3).

**Figure 4:**
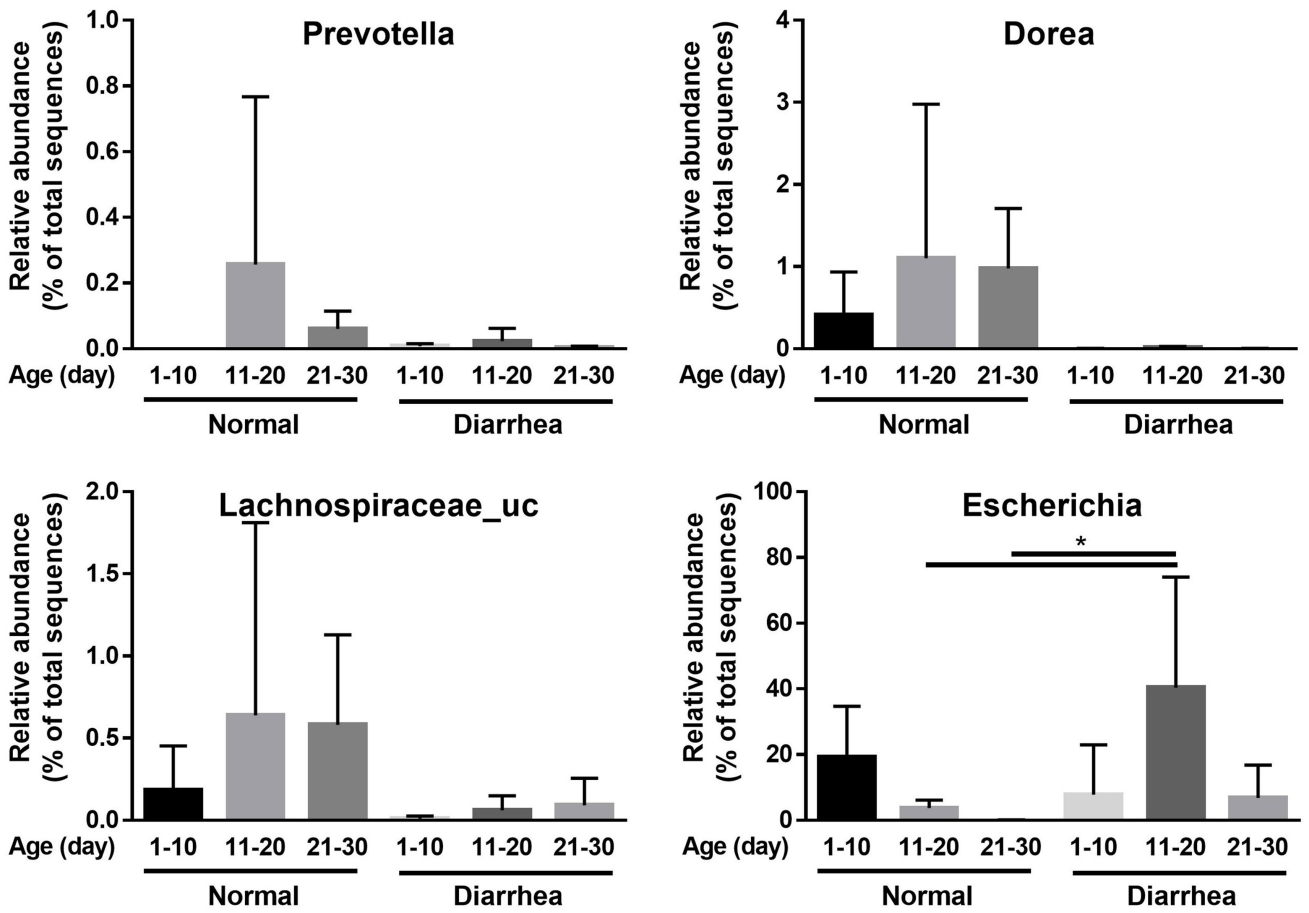
Comparison of relative abundance of microorganisms showing differences between normal calves (Normal) and calves with diarrhea (Diarrhea) at the genus level.

In addition, an increase in *Firmicutes* and a decrease in *Proteobacteria* were observed in the diarrhea (1–10 days) group compared to the normal (1–10 days) group, whereas a decrease in *Firmicutes* and an increase in *Proteobacteria* were observed in the diarrhea (11–20 days) group compared to the normal (11–20 days) group. However, no significant differences were observed, as the interindividual differences were large (Figure 3). In addition, when the intestinal microbiota that showed differences at the phylum level were analyzed at the genus level, *Prevotella*, *Dorea*, and *Lachnospiraceae_uc* decreased in calves with diarrhea, and *Escherichia* significantly increased in diarrhea (11–20) compared to normal (11–20) and normal (21–30) (Figure 4).

### 3.5 Selection of key microorganisms

**Figure 5:**
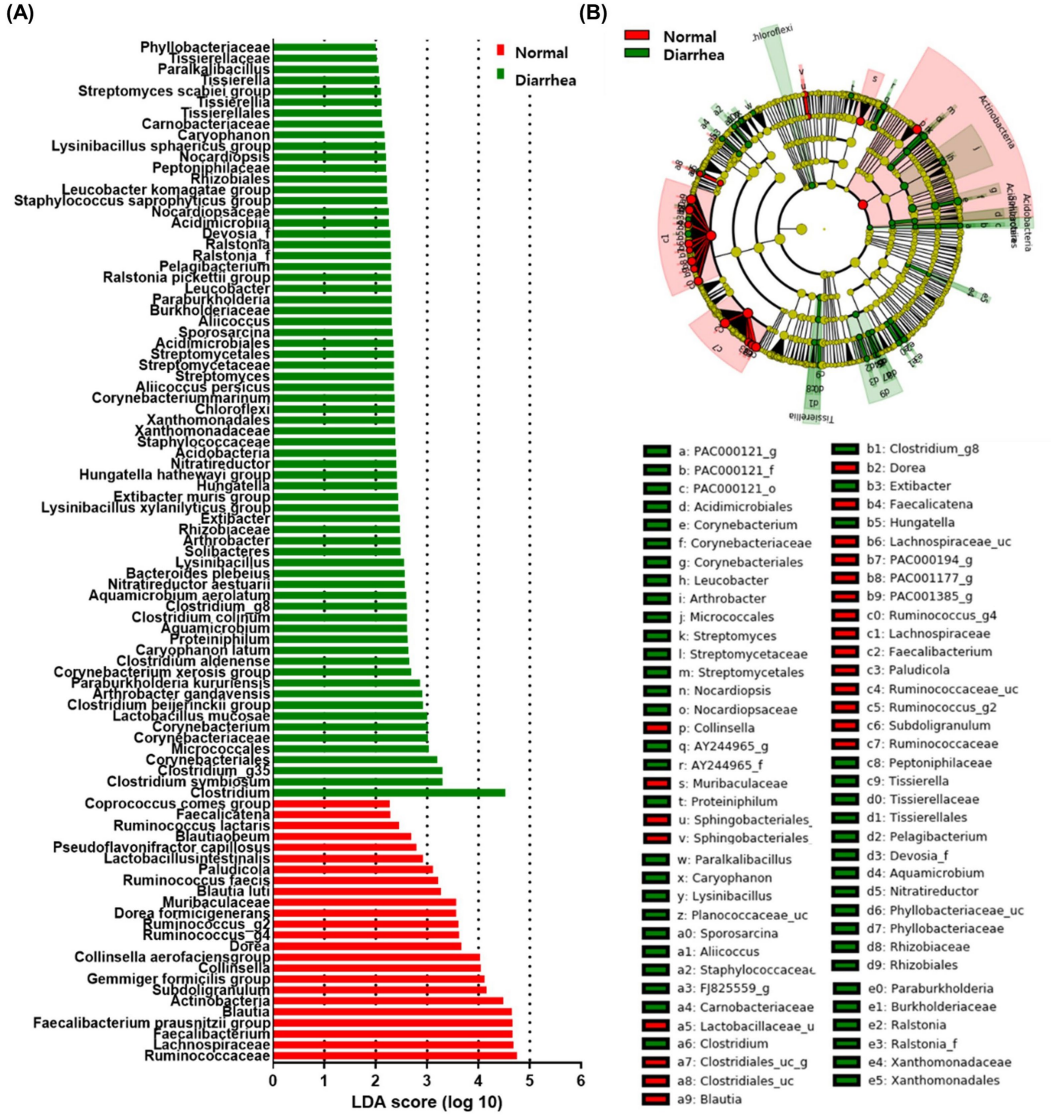
Linear discriminant analysis effect size (LEfSe) analysis of normal calves (Normal) and calves with diarrhea (Diarrhea) within 30 days of age. Results of linear discriminant analysis (LDA) analysis **(A)** and cladogram analysis **(B)** at the species level.

For early screening of diarrhea, LEfSe analysis was performed to identify intestinal microbiota related to diarrhea. As a result, depending on the presence or absence of diarrhea, 3 phyla (*Actinobacteria*, *Acidobacteria*, and *Chloroflexi*), 3 classes (*Solibacteres*, *Acidimicrobiia*, and *Tissierellia*), 8 orders (*Corynebacteriales*, *Micrococcales*, *Staphylococcaceae*, *Xanthomonadales*, *Streptomycetales*, *Acidimicrobiales*, *Rhizobiales*, *Tissierellales*), 15 families (*Ruminococcaceae*, *Lachnospiraceae*, *Muribaculaceae*, *Corynebacteriaceae*, *Rhizobiaceae*, *Xanthomonadaceae*, *Streptomycetaceae*, *Burkholderiaceae*, *Ralstonia_f*, *Devosia_f*, *Nocardiopsaceae*, *Peptoniphilaceae*, *Carnobacteriaceae*, *Tissierellaceae*, and *Phyllobacteriaceae*), and 31 genera (*Faecalibacterium*, *Blautia*, *Subdoligranulum*, *Collinsella*, *Dorea*, *Ruminococcus_g4*, *Ruminococcus_g2*, *Paludicola*, *Faecalicatena*, *Clostridium*, *Clostridium_g35*, *Corynebacterium*, *Proteiniphilum*, *Aquamicrobium*, *Clostridium_g8*, *Lysinibacillus*, *Arthrobacter*, *Extibacter*, *Hungatella*, *Nitratireductor*, *Streptomyces*, *Sporosarcina*, *Aliicoccus*, *Paraburkholderia*, *Leucobacter*, *Pelagibacterium*, *Ralstonia*, *Nocardiopsis*, *Caryophanon*, *Tissierella*, and *Paralkalibacillus*) were identified (Figure 5). Furthermore, at the species level, increases in 11 species (*Faecalibacterium prausnitzii* group, *Gemmiger formicilis* group, *Collinsella aerofaciens* group, *Dorea formicigenerans*, *Blautia luti*, *Ruminococcus faecis*, *Lactobacillus intestinalis*, *Pseudoflavonifractor capillosus*, *Blautia obeum*, *Ruminococcus lactaris*, and *Coprococcus comes* group) as well as 22 species (*Clostridium symbiosum*, *Lactobacillus mucosae*, *Clostridium beijerinckii* group, *Arthrobacter gandavensis*, *Paraburkholderia kururiensis*, *Corynebacterium xerosis* group, *Clostridium aldenense*, *Caryophanon latum*, *Clostridium colinum*, *Aquamicrobium aerolatum*, *itratireductor aestuarii*, *Bacteroides plebeius*, *Lysinibacillus xylanilyticus* group, *Extibacter muris* group, *Hungatella hathewayi* group, *Corynebacterium marinum*, *Aliicoccus persicus*, *Ralstonia pickettii* group, *Staphylococcus saprophyticus* group, *Leucobacter komagatae* group, *Lysinibacillus sphaericus* group, and *Streptomyces scabiei* group) were confirmed in normal calves and calves with diarrhea, respectively (Figure 5; Supplementary Figure 2). The microorganisms associated with diarrhea in calves were mainly known as harmful microorganisms.

**Figure 6:**
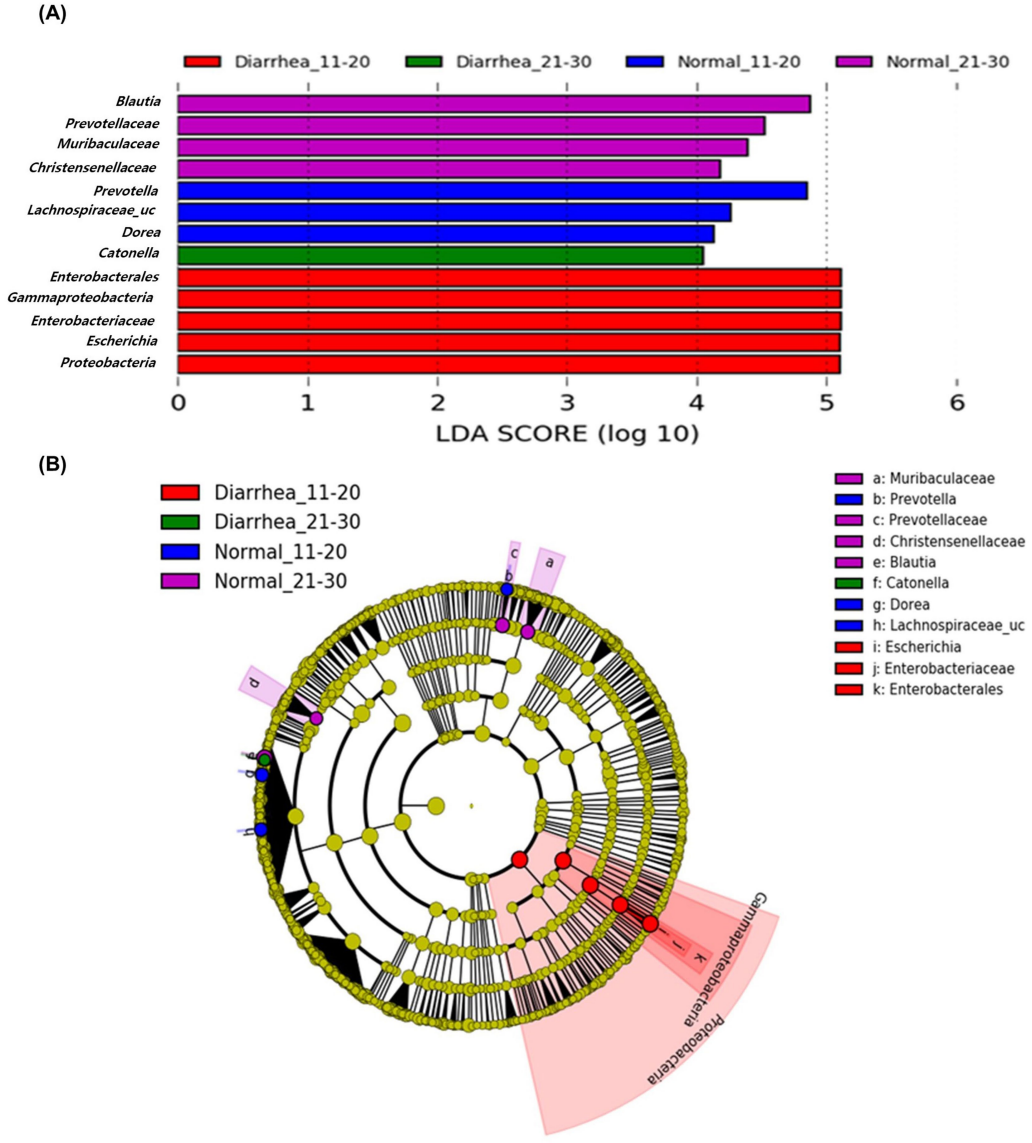
Linear discriminant analysis effect size (LEfSe) analysis results of the gut microbiome of calves aged 11–20 and 21–30 days according to age group and presence of diarrhea. Results of linear discriminant analysis (LDA) analysis **(A)** and cladogram analysis **(B)** at the genus level.

Analysis at the genus level according to age showed that there were no differences in the gut microbiome between the normal (1–10 days) and diarrhea (1–10 days) groups. However, *Prevotella* and *Lachnospiraceae_uc* in the normal (11–20 days) group, *Enterobacterales*, *Gammaproteobacteria*, *Enterobacteriaceae*, *Escherichia*, and *Proteobacteria* in the diarrhea (11–20 days) group, *Blautia*, *Provotellaceae*, *Muribaculaceae*, *Christensenellaceae*, and *Catenella* in the normal (21–30 days) group, and *Dorea* in the diarrhea (21–30 days) group showed significant differences (Figure 6). There were significant differences in the microorganisms that showed association among the groups, indicating that number of days and the presence of diarrhea in calf were significantly related to changes in the microbiome.

## 4 Discussion

The intestines of cattle contain a large and complex microbial community that interacts with the host to influence its health and gut microbiome structure. Analysis of the fecal microbiota can be used as an indicator of the gut microbiome (Zmora et al., 2018). In this study, the fecal microbiomes of neonatal Korean indigenous calves with diarrhea symptoms were analyzed to confirm significant changes. In addition, intestinal microorganisms that can be used as indicators of calf health were selected.

Previous studies have reported that *Firmicutes*, *Bacteroidetes*, and *Proteobacteria* account for most of the fecal microbiome during the growth stages of calves (Oikonomou et al., 2013; Kim et al., 2021; Malmuthuge et al., 2019; Meale et al., 2016). In the present study, the same microorganisms accounted for most of the gut microbiome. These microorganisms are abundant in the vagina and oral cavity of cattle, transmitted from mother to calf, and are abundant from birth (Laguardia-Nascimento et al., 2015). In addition, calves with diarrhea showed an increase in *Proteobacteria* and a decrease in *Bacteroidetes* and *Actinobacteria*. *Proteobacteria* is one of the groups of microorganisms that includes potentially harmful bacteria such as *E. coli* (Shin et al., 2015). As diarrhea occurs, the relative abundance of Firmicutes and *Proteobacteria* increases, thereby increasing the ratio of *Firmicutes*/*Bacteroidetes* in calves with diarrhea (Li et al., 2023). Therefore, the increase or decrease in these microorganisms can be used as an indicator of the imbalance in the gut microbiome of Korean indigenous calves due to diarrhea.

The anaerobic microorganisms *Faecalibacterium prausnitzii*, *Gemmiger formicilis*, and *Collinsella aerofaciens* were correlated with normal calves and were observed to decrease with the occurrence of diarrhea. This is consistent with previously reported changes in the gut microbiome of calves with diarrhea (Jang et al., 2019; Gomez et al., 2022; He et al., 2022; Li et al., 2023). Anaerobic microorganisms are involved in intestinal fermentation to produce short-chain fatty acids, and a decrease in these microorganisms is a characteristic of diarrhea (Cui et al., 2023; Li et al., 2018; Morrison and Preston, 2016). Therefore, a decrease in these anaerobic microorganisms can be used as a biomarker for diarrhea in Korean indigenous calves. The gut microbiome diversity of normal calves and calves with diarrhea showed a significant difference in Simson index, one of the evenness, which indicates evenness. The Simson index is the probability that two randomly selected individuals belong to the same species, and the closer it is to 1, the lower the evenness. The Simson index was significantly higher in calves with diarrhea, which shows that diarrhea changes the relative abundances of gut microbiome, causing imbalance in the gut microbiome. In addition, PCoA analysis results showed that calves with diarrhea showed a difference in the distance between the intestinal microbiota community and normal calves. This also shows that the gut microbiota of calves with diarrhea is different from that of normal calves. However, at the phylum level, the microbial community of normal calves did not show significant differences with age, whereas changes in *Firmicutes* and *Proteobacteria* occurred in calves aged 1–10 and 11–20 days as diarrhea occurred. These results confirm that the gut microbiome of Korean indigenous calves within 30 days of birth showed greater differences based on the presence or absence of diarrhea than based on age. However, because the environment and pathogen type can significantly affect the intestinal microbiota, additional research is needed to determine the specific causes (Jang et al., 2019; Fu et al., 2023; Li et al., 2022). In addition, previous studies have reported that the structure of the gut microbiome differs depending on the time of fecal collection (within 24 or 48 h after the onset of diarrhea in calves) (Li et al., 2023). Similarly, in Korean indigenous calves, research is needed on the gut microbiome in the early stage of diarrhea, when the most active changes occur, and it is necessary to standardize the time of feces collection from the occurrence of diarrhea.

Diarrhea is a serious disease in neonatal calves that slows growth and, in severe cases, can even lead to death. Therefore, it is important to predict and treat diarrhea early. However, because diarrhea is caused by various reasons, it is difficult to predict diarrhea before clinical symptoms appear. Biomarkers are indicators that show statistical differences between groups and are used for early diagnosis and prediction of diseases (Chang et al., 2022). LEfSe analysis of the gut microbiome of Korean indigenous calves identified biomarkers with significant differences depending on the presence or absence of diarrhea. The results confirmed that *Faecalibacterium*, *Blautia*, *Subdoligranulum*, *Ruminococcus*, *Clostridium*, *Corynebacterium*, *Arthrobacter*, *Dorea*, and *Escherichia* were associated with diarrhea. That is, as in previous studies, beneficial microorganisms decrease whereas harmful microorganisms, known as pathogenic microorganisms, increase (Zeineldin et al., 2018; Carroll et al., 2012; Brunkwall et al., 2021; Xin et al., 2021; Chuang et al., 2022; Fecteau et al., 2016; Zhu et al., 2024; Ma et al., 2020). This suggests that these microorganisms identified as biomarkers could be used as predictors to predict diarrhea in calves. In addition, as a result of the LDA analysis, no microorganisms showing differences were identified in the diarrhea (1–10 days) group; however, *Enterobacterales*, *Gammaproteobacteria*, *Enterobacteriaceae*, *Escherichia* and *Proteobacteria* were identified in the diarrhea (11–20 days) group whereas *Dorea* was identified in the diarrhea (21–30 days) group. Therefore, we confirmed that changes in the gut microbiome due to an increase in harmful bacteria were related to diarrhea. It is necessary to consider the age of Korean indigenous calves to diagnose and predict diarrhea using the gut microbiome. In addition, selecting key microorganisms with high diagnostic accuracy among diarrhea-related microorganisms using the AUC-ROC curve will be helpful in predicting the occurrence of diarrhea. Gut microbiome testing using feces is easy to collect samples, but it cannot be used directly on the farm and the testing cost is still high. Therefore, it is realistically difficult to conduct screening for all individuals, but it is clear that the analysis of the intestinal microbiome of calves through feces will be helpful in predicting diarrhea. In this study, it was confirmed that harmful bacteria were highly related to diarrhea in Korean indigenous calves, and therefore, in order to prevent diarrhea, it is necessary to establish an intestinal environment that can suppress the growth of harmful bacteria in the intestines of calves. Supplying direct fed microbials such as probiotics helps to suppress the colonization of harmful bacteria in the intestines of calves and further improves the intestinal environment (McAllister et al., 2011).

There are still some limitations in this study. The intestinal microbiota changes depending on the environment, and especially neonatal calves are in a period of rapid intestinal microbiota change. Since we used 3–5 calves per group raised on the same farm, generalization to all Korean indigenous calves may be limited. However, the intestinal microbiota of Korean indigenous calves has rarely been reported so far, and therefore the results of this study on the changes in the intestinal microbiota due to diarrhea in Korean calves are thought to be of sufficient value in Korea. In calves, diarrhea is caused by a combination of factors such as the presence of pathogens, the immune status of the calf, and insanitary environments. Another limitation is that the gut microbiota of calves with diarrhea was not analyzed before the onset of diarrhea, and the cause of the calves’ diarrhea was not identified. Therefore, comparing the intestinal microbiota before and after diarrhea in calves according to each cause will clearly explain the changes in the intestinal microbiota due to diarrhea in Korean indigenous calves.

In conclusion, this study confirmed that diarrhea significantly affects the intestinal microbiota of Korean indigenous calves. Diarrhea reduces the diversity of the intestinal microbiota of calves and increases the proportion of harmful microorganisms, creating an unbalanced intestinal environment. In addition, newborn calves (1–10) showed significant differences in the evenness index in diarrhea, but the microbiota was still unstable, so the occurrence of diarrhea did not show significant differences in the microbiota from normal calves. However, calves aged 11–30 days showed differences in the intestinal microbiota structure and microorganisms related to diarrhea depending on age. Therefore, predict and prevention of diarrhea in neonatal calves would be helpful for growth and health management of calves. This study revealed changes in the intestinal microbiota following diarrhea in Korean calves. This may help establish future research directions for the intestinal microbiota of calves and methods for preventing diarrhea in calves through intestinal microbiota management.

## Data Availability

The raw data supporting the conclusions of this article will be made available by the authors, without undue reservation.

## Footnotes

^1^ https://www.mothur.org

^2^ www.ezbiocloud.net/

^3^ https://huttenhower.sph.harvard.edu/galaxy/

## Supplementary material

Supplementary Figure 1 Rarefaction curve of fecal samples collected from 25 Korean indigenous calves.

Supplementary Figure 2 Comparison of relative abundance of microorganisms showing differences between normal calves (Normal) and calves with diarrhea (Diarrhea) at the species level.
