# Supplementary figures and images for: Changes in the gut microbiome due to diarrhea in neonatal Korean indigenous calves
Source: Front Microbiol. 2025 Mar 5;16:1511430. doi: 10.3389/fmicb.2025.1511430 (PMC11921620; doi:10.3389/fmicb.2025.1511430)

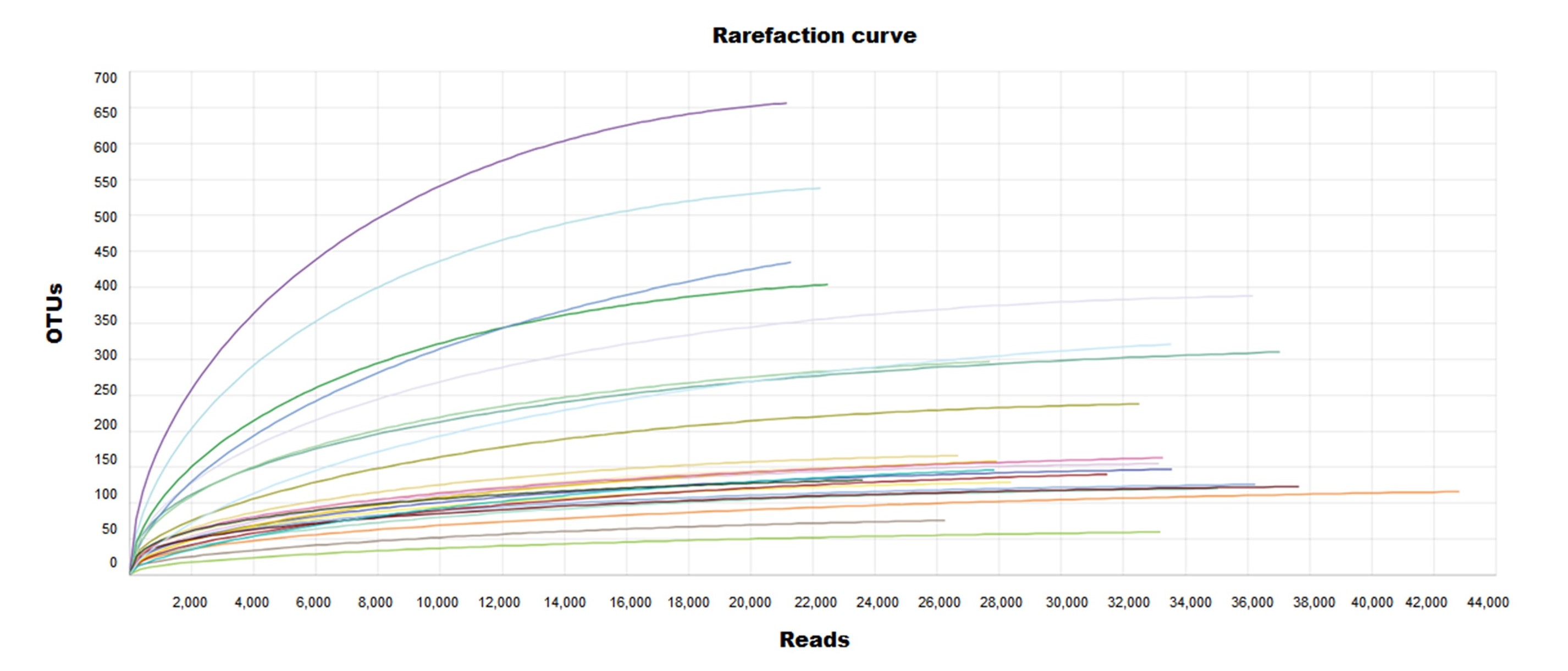

Supplement: Supplementary Figure 1 — Rarefaction curve of fecal samples collected from 25 Korean indigenous calves. [file Image_1.jpeg]

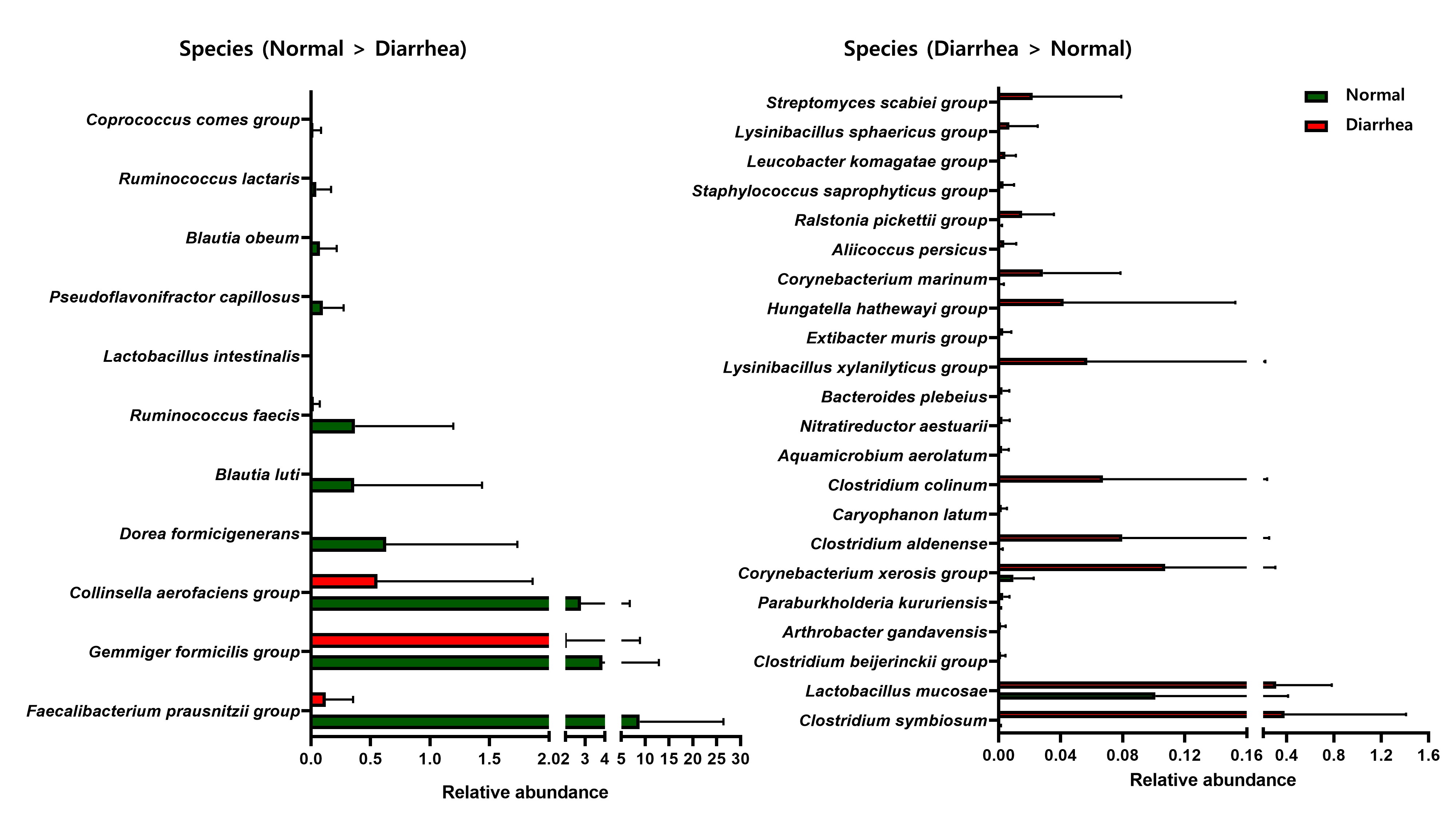

Supplement: Supplementary Figure 2 — Comparison of relative abundance of microorganisms showing differences between normal calves (Normal) and calves with diarrhea (Diarrhea) at the species level. [file Image_2.jpeg]
